# The Double Historicity of Sex and Gender

**DOI:** 10.1007/s10508-025-03308-x

**Published:** 2025-09-29

**Authors:** Alex Thinius

**Affiliations:** Department of Philosophy, Universiteit van Amsterdam, Oude Turfmarkt 141-143, 1012 GC Amsterdam, The Netherlands

How many sexes are there? How many genders? Answering these questions is not just challenging: It is about as silly as trying to determine how many kinds of stories or how many species there are: Where? And when? And, most importantly, in what sense? Instead, I suggest taking a closer look at the double historicity of phenomena and concepts of sex/gender: Research concepts of sex and gender are context-dependent, plural, and historically dynamic; and so are the phenomena that these concepts refer to. For reasons of both scientific accuracy and justice, understanding the double historicity of sex/gender transforms the “How many are there” questions. Instead of absolutist single-answer approaches of the “There are x number of sexes/genders” style, it demands a research ethos that involves contextual awareness regarding concepts and phenomena; reflective distance from one’s own analytical research concepts and aims; and epistemic humility.

## “Sex” and “Gender” Are Said in Many Ways

The referents of “sex” and “gender” are historically complex. They involve contextual variation, cross-level interaction, entanglement, dynamicity, and plurality of most (if not all) dimensions of sex and gender (Fausto-Sterling, 2020; Prum, 2023). Research on interactions between cis/trans-ness, sex diversity, and (post-)colonial categorization practices deepens this knowledge (Driskill, 2010; Dutta, 2012, 2025; Epple, 1998; Nzegwu, 2006; Snorton, 2017).

Likewise, research concepts of sex and gender are contextually variable and historically changing. For example, in some research contexts, “gender” refers to a kind of social position that binds a power status to the particular concept of sex held in that social setting (Haslanger, 2012). Other research contexts use “gender” for those patterns of social practice (connecting socio-cultural symbols, roles, norms, actions, materials) with which some societies assemble and configure the “reproductive arena” of making, having, and nursing babies, and associated domains (Connell, 2006). Other contexts use “gender” to refer to a “combination of personality traits” (Zucker, 2025). Yet others use “gender” to refer to a person’s self-concept or self-identification with a gender category (Steensma et al., 2013).

Similarly for “sex”: Some concepts of sex aim at cataloging kinds of traits (e.g., genitals, gonads, genetics), whereas others aim at causal relationships (e.g., “is not causally shaped by social processes”) (cf. Heinämaa, 2012). Then again, some aim at categorizing kinds of individuals (“This robin is a male”) or qualities of individuals (“This robin is male”). Others aim at characterizing reproductive strategies at species level (“The species of robins reproduces by mixing gametes”). Moreover, sometimes, sex is defined along one dimension, e.g., in terms of genetics (Pfaus et al., 2025) or in terms of reproductive strategy (Goymann et al., 2022; Roughgarden, 2013). In other contexts, “sex” is conceptualized to include multiple dimensions (Joel, 2012; McLaughlin et al., 2023; Rehmann-Sutter et al., 2023).

I believe that each conception of sex and gender has its specific epistemic utility and limitations. For example, take spectrum concepts of gender and sex (Monro, 2005; Tanaka & Tachibana, 2022). Their strength lies in grasping continuous differentiation beyond, across, or within categories, as well as providing coordinates for locating individuals and their transformations across a gradient. Yet, for grasping the change of gender categories and properties, other concepts, models, or cognitive metaphors are needed (Thinius, 2024).

Given the widespread confusion of different sex/gender operationalizations and concepts (Albert & Delano, 2022), it is imperative to be precise about actual reference, limits, and strengths of each concept, without setting any one of them in stone. Sober methodologies benefit from fine-grained distinctions between what was once lumped together as “the sexes.” One such approach is “sex contextualism” (Richardson, 2021). However, one has to ask how beneficial the concept of sex is in a concrete research project, given the risk of proliferating mistaken equations between different phenomena. Replacing the concept of sex with more precise concepts might be reasonable, at least in some contexts (Watkins & DiMarco, 2025). For example, depending on research context, “sex” may be replaceable with “gamete size,” “testosterone level,” “weight differences,” “educational differences,” “occupation,” “gender identity,” “sex assigned at birth,” “care burden,” or “rate of medication use” (for the latter, see Boulicault et al., in press).

## Struggles for Recognition of Sex/Gender Complexity

There is an interesting historical dynamic between pluralism in academic sex/gender concepts, social recognition of plurality in people, and negotiations with the idea that sex is binary. Already around the year 1900, evidence challenged the idea of a fixed and binary order among the sexes, leading to conceptual renegotiations. The following example from Germanophone academia reveals a pair of strategies in the ensuing conceptual negotiations: preserving the idea that sex (including gender and sexuality) is binary versus aiming to overcome it.

On the one hand, some binary concepts of the sexes explained away this variation: If they are not real, maybe, male and female are platonic ideas like two “Substanzen” (Weininger, 1908, pp. 9–12), where only some people—white and in accordance with bourgeois ideals—counted as really materializing these ideal types (Hinchy, 2019; Schuller, 2018; Sengoopta, 1992). On the other hand, there were prominent anti-essentialist and pluralist responses to such typological thinking (Leck, 2012; Stern & Szabados, 2004). These moved from concepts of “third sex/gender” (“drittes Geschlecht”) to sexual series and intermediaries (Frey, 1898; Hirschfeld, 2015), next to developmentalist approaches (Freud, 1989). This created significantly more recognition for human diversity, without, however, overcoming the racialization of sex/gender/sexuality types (“Vollweib,” “Vollmann,” “wahre Homosexualität”) as reference points (Çetin et al., 2016; Gilman, 1992; Mehlmann, 2006).

These more pluralist concepts, along with related knowledge and archives, were not refuted by using academic counterevidence or new insight: First, they were crushed by political interventions, censorship, and police repression since the first year of German fascist government, treating deviations from two ideals of racialized sex/gender/sexuality types not as evidence for diversity but as criminal, pathological, or otherwise unworthy forms of life (Fuller & Owen, 2022; Haeberle, 1981). Secondly, a conceptual model for heredity gained influence in academia: neo-Mendelianism. From the conceptual needs of this theory, it seemed a negotiable trade-off to bracket the complexity of sex/gender in actual organisms for an idealization of a binary between XX and XY chromosomes (Richardson, 2013).

## Recognizing Complexity at the Price of Re-Affirming the Sex Binary

Dimension after dimension was distinguished from the mid-twentieth-century concept of “sex.” For example, recognizing sexual orientation as its own phenomenon allowed it to be conceptualized on a seven-point scale, bypassing the conceptual straightjacket of a binary sex trait (Drucker, 2014). Gender self-concept, gender identity, and gender personality traits came to be recognized as psychological phenomena with an ambiguous relation to sex. Moreover, studying social and cultural dimensions associated with sex benefitted from being recognized as distinct phenomena, increasingly called “gender.” At least by the 2000s, this allowed many to conceptualize dimensions of gender as non-pathologically plural beyond the binary (Fausto-Sterling, 2020; Monro, 2005; Reilly, 2019).

Similarly for research into the remaining dimensions of sex, in the mid-twentieth century, genetic sex was supposed to guarantee the binary character of sex. Yet soon, this job had to be reassigned: to hormones, gonads, brains. By now, it is clear that these traits are useless for grounding a clearcut binary, let alone a definition of sex valid for non-human species as well as humans (Roughgarden, 2013). At least one percent of the human population cannot be classified as either male or female on these kinds of definitions, prompting researchers to add at least the dependent category of intersex (Joel, 2012). Similarly, recent articulations of a binary definition of sex, such as Goymann et al. (2022), call it a “widespread misconception … that the definition of the biological sex is based on chromosomes, genes, hormones, vulvas, or penises. … [For example, h]omozygous sex chromosomes are indeed reliable operational criteria to predict a biological sex in mammals and in birds, but they predict different sexes in these two taxa” (p.4).

Throughout these conceptual negotiations, there is a pattern: A phenomenon is accepted to be non-binary on the condition that it is recognized as not “sex itself” (for example, as merely a sex-related trait or as something entirely different). This improves scientific knowledge and generates more social recognition for gender/sex minorities. However, it comes at the price of reaffirming that sex itself, whatever it is, is categorically binary, followed by a new search for the truly definitional binary dimension of sex.

## The Double-Edged Sword of the Anisogamy Definition

The anisogamy definition of sex is currently seen as the most promising home for the idea that something related to what people think of as “sex” and “gender” is binary. Reducing the definition of sex to this one dimension allows, first, recognition of the non-binary complexity of all other dimensions of the nineteenth-century concept of “sex”: genitals, gonads, genetics, the many dimensions of gender, sexual orientation, and their relationship to people’s brains. Secondly, it may be epistemically beneficial to define sex as the reproductive strategy of mixing two gametes of significantly different size, for instance in abstract species-level models of sexual or social selection (Roughgarden, 2015).

There we go: Two gametes mix. Small and large may be vague categories, but fairly binary. Yet, on this definition, sex is a species-level property, not a property of mating types, individuals, or their traits. Once mating types, sex roles, and organisms enter the picture, with their traits and processes, things appear “messy” again: Anisogamy does not simply sort organisms into males and females (Griffiths & Spencer, 2025; Roughgarden, 2013).

Anisogamy definitions of sex have recently traveled and proliferated across scientific disciplines, academic fields, law, and policy. Yet insight into their severe limitations does not cross these borders equally. This way, in effect, anisogamy definitions are inaccurately used to bolster the mistaken belief that sex, and sometimes even gender, is differentiated as a categorical binary among (human) individuals, give or take some marginalized exceptions.

## The Gender Agenda of Antidemocratic Politics

Especially under ideal democratic conditions, extra attention to research contexts (see above), careful science education, and dedicated public outreach might prevent this kind of misinformation and abuse. But this is not the world we live in.

“The Government’s standpoint is that people are born either male or female,” said the spokesperson of the Hungarian prime minister in 2018 (Kent & Tapfumaneyi, 2018). Research on sex and gender was censored in Hungary, as academic freedom eroded so rapidly that the Central European University was forced to flee the country (Thorpe, 2020). Bringing “death by a thousand cuts” to democracy (Rohlfing & Wind, 2023), Hungary’s transformation from democracy to “zombie democracy” and autocracy instrumentalized and fostered misinformation, censorship, and outrage specifically around gender (next to its other main platform of immigration anxieties). In the USA in 2025, the Republicans under Trump copied the autocratic leader of Hungary almost verbatim (Wendling & Epstein, 2025).

Pushing the idea of binary sex determinism and stirring fears of pluralist and dynamic approaches to sex/gender serve two aims for the New Right: On the one hand, for example in Europe, throughout the past 15 years, networks of religious and political actors were genuinely serious about pushing illiberal and patriarchal sex/gender/sexuality politics based on binary sex determinism, under the slogan “restoring the natural order” (Datta, 2018). On the other hand, “gender” is also used as a “symbolic glue” (Kovats & Poim, 2015) in a merely strategic way to forge possibly surprising new alliances between groups often opposed to each other and to far-right political visions: across gender positionalities; across conservatives, right extremists, and some leftists; as well as across ethnic and religious social strata. Misinformation and symbolic fearmongering against sex/gender plurality are being used to shift public discourses toward a new version of common sense. Somewhat heterogeneous in its ideologemes and politics, this strategy centers illiberalist, anti-democratic, and pro-autocratic visions and policies of the New Right (Dietze & Roth, 2020).

This situation raises the stakes for embracing binary concepts of sex and gender even in academic research (Sudai et al., 2022). Does their restricted utility for some specific research contexts outweigh the epistemic risk of their biased travels and instrumentalization?

## Doing Justice to the Phenomena, When the Phenomena Are People

For over a century, much academic research in the West has been devoted to finding the true nature of what the nineteenth century used to call the sexes. This led to solid insight: What people typically refer to as “sex” and “gender” is not categorically binary; socio-cultural arrangements play an important causal role in sex and gender development; and hierarchies between so-positioned men, women, and sex/gender minorities are due to a socio-historical situation. Yet, the scientific quest for the golden nuggets of manhood and womanhood has not been called off, producing evermore binary sex difference hypotheses.

The under-complex epistemic inadequacy of binary and basically binary conceptual research tools makes (human) variation appear as anomalous. Sometimes, it is even (unintentionally) lumped together with noise and measurement error, impeding the recognition of counterevidence to a hypothesis (Thinius & Trappes, 2024). This combines with entrenched social causal effects, where binary sex/gender/sexuality concepts “create messy individuals” in the double sense of yielding messy scientific pictures and fueling social marginalization. Put more poetically, one may say that binary sex/gender categories are continuously haunted by the ragtag crowd of phenomena that simply cannot be recognized through them. Somewhat like suppressed ghosts, wrongfully pushed into the shadows and to social death, these so-called anomalies, idiosyncrasies, and pathologies resulting from over-simplistic sex/gender categories keep returning to demand recognition.

Idealized (binary) operationalizations and models of some dimension of sex or gender can of course have epistemic merit—for example, the anisogamy definition seems a useful and maybe epistemically benign simplification for specific idealized species- or taxa-level models. However, we should be extra careful to examine their utility and limitations, because of the massive binary sex essentialist bias among entrenched research practice and broader audiences, which are easily instrumentalized and further fueled by authoritarian politics.

When research goes where evidence and the “phenomena” are taking it, it finds that the coarse-grained categories of “man” and “woman” or “male” and “female” do not even begin to capture the complexity of sex/gender/sexuality, not even when dependent “rest” categories of intersex and trans* are added. Especially regarding gender, as the future is open and social contexts are multilayered, we cannot say how many gender categories humans will come up with to make more and less adequate sense of variation regarding sex/gender dimensions. Science for a less repressive society would include more contextual awareness, conceptual distance, and epistemic humility. It would work toward concepts and practices that foster recognition and ability for equal participation for everybody.

## Data Availability

Not applicable.
